# Reduced body-image disturbance by body-image interventions is associated with neural-response changes in visual and social processing regions: a preliminary study

**DOI:** 10.3389/fpsyt.2024.1337776

**Published:** 2024-03-06

**Authors:** Yumi Hamamoto, Kentaro Oba, Ryo Ishibashi, Yi Ding, Rui Nouchi, Motoaki Sugiura

**Affiliations:** ^1^ Institute of Development, Aging and Cancer, Tohoku University, Sendai, Japan; ^2^ Department of Psychology, Northumbria University, Newcastle upon Tyne, United Kingdom; ^3^ Japan Society for the Promotion of Science, Tokyo, Japan; ^4^ School of Medicine, Tohoku University, Sendai, Japan; ^5^ International Research Institute of Disaster Science, Tohoku University, Sendai, Japan

**Keywords:** body image, fMRI, mirror exposure, mental imagery, intervention

## Abstract

**Introduction:**

Body-image disturbance is a major factor in the development of eating disorders, especially among young women. There are two main components: perceptual disturbance, characterized by a discrepancy between perceived and actual body size, and affective disturbance, characterized by a discrepancy between perceived and ideal body size. Interventions targeting body-image disturbance ask individuals to describe their own body without using negative expressions when either viewing it in a mirror or imagining it. Despite the importance of reducing body-image disturbance, its neural mechanisms remain unclear. Here we investigated the changes in neural responses before and after an intervention. We hypothesized that neural responses correlated with the degree of body-image disturbance would also be related to its reduction, i.e., a reduction in perceptual and affective disturbances would be related to changes in attentional and socio-cognitive processing, respectively.

**Methods:**

Twenty-eight young adult women without known psychiatric disorders underwent a single 40-min intervention. Participants completed tasks before and after the intervention, in which they estimated their perceived and ideal body sizes using distorted silhouette images to measure body-image disturbance. We analyzed the behavioral and neural responses of participants during the tasks.

**Results:**

The intervention did not significantly reduce body-image disturbance. Analysis of individual differences showed distinct changes in neural responses for each type of disturbance. A decrease in perceptual disturbance was associated with bodily visuospatial processing: increased activation in the left superior parietal lobule, bilateral occipital gyri, and right cuneus. Reduced affective disturbance was associated with socio-cognitive processing; decreased activation in the right temporoparietal junction, and increased functional connectivity between the left extrastriate body area and the right precuneus.

**Discussion:**

We identified distinct neural mechanisms (bodily visuospatial and socio-cognitive processing) associated with the reduction in each component of body-image disturbance. Our results imply that different neural mechanisms are related to reduced perceptual disturbance and the expression thereof, whereas similar neural mechanisms are related to the reduction and expression of affective disturbance. Considering the small sample size of this study, our results should be regarded as preliminary.

## 1 Introduction

Body-image disturbance is characterized by distorted perceptions and attitudes related to one’s body, such as a tendency to overestimate one’s size, negative self-evaluation of one’s body, and an excessive focus on body weight and shape during self-evaluation. It is recognized as a core feature of eating disorders (1, 2). Developing symptoms of an eating disorder is most common among adolescent females; thus, research on the relationship between body-image disturbance and eating disorders has focused mainly on this demographic. Recent studies have revealed that a non-clinical community sample also experience body-image disturbance as strong as those of individuals with eating disorders (3–6). Furthermore, individuals with relatively high body-image disturbance are more likely to develop symptoms associated with eating disorders (7, 8). Several studies have revealed that reduced body-image disturbance is associated with an improved prognosis of individuals with eating disorders (9–12). Therefore, reduction in body-image disturbance is crucial for both the prevention and treatment of eating disorders.

When considering strategies for reducing body-image disturbance, it is crucial to recognize the two components of body-image disturbance, namely perceptual disturbance and affective disturbance, each of which involves distinct psychological and neurological processes. Perceptual disturbance involves distorted perception of one’s body size and shape, such as overestimating one’s body size (5, 13–15). This component is quantified as the discrepancy between one’s perceived and actual body size (perceived–actual discrepancy) (3–6, 15). Previous studies have associated perceptual disturbance in association with attentional bias (4, 16, 17). These studies revealed that individuals with greater perceived–actual discrepancy exhibited specific fixation patterns, such as focusing on the waist. Neurological studies have demonstrated that perceptual disturbance is related to brain activation in bodily visual and attentional processing regions, such as the extrastriate body area, inferior parietal lobule, and anterior cingulate cortex (3, 6, 18–21). Notably, we found that perceived–actual discrepancy was correlated with increased activation of the left anterior cingulate cortex and functional connectivity between the left extrastriate body area and the right anterior insula when participants were estimating their own body size (6), implying that perceptual disturbance was related to attentional processing rather than visual processing only (22, 23).

On the other hand, affective disturbance involves disturbances in attitudes and feelings toward one’s body, such as excessive dissatisfaction therewith (13). Affective disturbance, observed in almost all eating disorders, is related to the excessive influence of weight and shape on self-esteem, a core psychopathological symptom (2, 9). This component is quantified as the discrepancy between one’s perceived and ideal body size (perceived–ideal discrepancy) (3–6, 15). Previous studies found that affective disturbance was associated with body-image concerns (5, 15, 24, 25). These studies revealed that the degree of affective disturbance was correlated with eating disorder inventory scores related to body-image concerns, such as body dissatisfaction. Neurological studies have demonstrated that affective disturbance is related to brain activation in emotional and socio-cognitive processing regions, such as the insula, amygdala, precuneus, and temporoparietal junction (3, 6, 20, 26–29). In particular, we revealed that perceived–ideal discrepancy correlated with an increase in activation of the right temporoparietal junction and decrease in functional connectivity between the left extrastriate body area and the right precuneus when participants were estimating their ideal body size (6). Considering that these regions are related to socio-cognitive processing, such as thinking about others (30, 31), these neural responses potentially reflect the influence of social pressure on the establishment of one’s ideal body. These psychological and neurological differences between the two types of disturbance imply that distinct neural mechanisms are also involved in reducing each type of body-image disturbance.

However, the neural mechanisms underlying the reduction in each type of body-image disturbance remain unclear. Considering the various psychological processes related to the development and maintenance of eating disorders and body-image concerns, neural responses may differ between the expression and reduction of body-image disturbance (9, 32, 33). Although it is crucial to understand the neural mechanisms related to the reduction in body-image disturbance, no previous study has directly investigated neural responses related to the reduction in each type of body-image disturbance. Previous studies have focused mainly on individuals with eating disorders and examined changes in neural responses when looking at body images before and after treatment (34, 35). These studies revealed increased activation in the extrastriate body area after treatment, which should be related to a reduction in body-image disturbance. However, these studies did not differentiate between the two components of body-image disturbance, and more importantly, they did not evaluate changes in body-image disturbance before and after treatment. Additionally, the findings from these studies are inconsistent with our previous finding that neural responses were correlated with the degree of each type of body-image disturbance (6). Therefore, it is plausible that the reported neural changes were not directly related to reduced disturbance. Moreover, the identified neural mechanisms can facilitate the development of effective novel treatments. Similar to other psychiatric disorders, interventions targeting neural responses may be effective (36–38). Several non-invasive strategies can be used to manipulate neural responses, such as neurofeedback (39) and transcranial magnetic stimulation (40). In real-time neurofeedback, individuals monitor and attempt to control their neural responses. On the other hand, in repetitive transcranial magnetic stimulation, a specific brain region related to psychopathological symptoms is activated or deactivated using a repeated magnetic pulse. When applied for reduction of body-image disturbance, these approaches target the underlying neural responses, providing novel and effective prevention and treatment strategies for eating disorders. To reveal the neural mechanisms related to the reduction in each type of disturbance, it is necessary to examine brain activation before and after interventions designed to reduce each type of body-image disturbance and investigate neural responses associated with a decrease in each disturbance type.

Two interventions, mirror exposure and mental imagery, have shown promise in reducing types of body-image disturbance, including body dissatisfaction, which is associated with affective disturbance (41–43). These interventions can also decrease attentional bias by diverting attention from specific body parts, implying that they can also reduce perceptual disturbances (4, 43–45). Both interventions encourage individuals to avoid negative expressions about their bodies, such as “fat”. Although mirror-exposure interventions have several variations, participants are typically instructed to look at a specific body part in a mirror, ensuring that they view each body part equally, and to describe them without using negative expressions (41, 42, 46–48). Compared to mirror exposure, mental imagery has fewer variations and involves describing one’s imagined body instead of looking at it. All studies of mental imagery require the participants to imagine a specific body part according to experimenter’s instructions and then describe it (42, 43). Compared to mirror exposure, mental imagery acts indirectly and is associated with a lower risk of aggravating negative feelings on exposure to one’s own body image (20, 43, 49). Redistribution of attention is considered a core feature of these interventions, which can address attentional bias toward specific body parts and reduce perceptual disturbance (4, 43–45). Additionally, several studies have demonstrated that prolonged viewing of unattractive body parts is associated with an increased likelihood of developing body dissatisfaction (5, 43, 50, 51). Mirror exposure and mental imagery share several common procedures that can potentially reduce each component of body-image disturbance. However, the effects of these interventions on both types of body-image disturbance can differ. Previous studies have demonstrated that both mirror exposure and mental imagery can reduce body dissatisfaction, but only mirror exposure was effective in reducing the frequency of negative thoughts and feelings of ugliness (42, 43). Considering the discrepancies between the aforementioned studies and others that demonstrated increased negative feelings on exposure to body image (20, 43, 49), the differences in the effects of the two interventions on each type of body-image disturbance, and the neural mechanisms underlying these effects, remain unclear.

The main objective of this study was to reveal the neural mechanisms underlying reductions in perceptual and affective disturbances. To achieve this, we recruited young women with body-image disturbance who had not been diagnosed with any psychiatric disorders, including eating disorders. We evaluated perceived–actual and perceived–ideal discrepancies before and after interventions, as well as functional magnetic resonance imaging (fMRI) data. We conducted multiple regression analysis to investigate the relationship between changes in neural responses and the reduction in each type of discrepancy. Based on our previous study (6), we hypothesized that neural responses related to the degree of each type of body-image disturbance would also be related to the reduction thereof. In other words, we expected that changes in attentional processing would be related to reduced perceptual disturbance, whereas changes in socio-cognitive processing would be related to reduced affective disturbance. A decrease in the perceived–actual discrepancy was expected to be associated with deactivation of the left anterior cingulate cortex and a reduction in functional connectivity between the left extrastriate body area and the left anterior insula. Similarly, a decrease in the perceived–ideal discrepancy was expected to be associated with deactivation of the right temporoparietal junction and an increase in functional connectivity between the left extrastriate body area and the right precuneus. Because the different procedures (i.e., looking at one’s body or imagining specific body parts) involved in the interventions can have distinct effects on body-image disturbance, we also investigated potential differences in neural responses related to the reduction in body-image disturbance associated with each intervention. Participants were randomly assigned to the mirror-exposure or mental-imagery group. There were no significant differences between the two groups in terms of behavioral factors (see Results for details). Therefore, this study evaluated group differences in neural mechanisms by examining two-factor interactions between intervention groups and neural-response changes related to body-image disturbance, rather than directly comparing neural-response changes between the two intervention groups.

## 2 Materials and methods

### 2.1 Ethical statement

The study protocol was approved (2020-1-1049) by the Ethics Committee of Tohoku University Graduate School of Medicine, Japan. Informed consent was obtained from all participants, and the study was conducted in accordance with the Declaration of Helsinki.

### 2.2 Study design

This fMRI study investigated the changes in neural responses related to the reduction in body-image disturbance before and after body-image interventions. Participants performed two tasks related to body-image disturbance in an MRI scanner before and after body-image interventions; the changes in the degree of body-image disturbance and neural responses were evaluated. We conducted a brief (1-day) intervention without any follow-up to determine acute changes in behavioral and neural responses. We examined the reduction in body-image disturbance at the collective and individual levels, assessing neural responses related to the reduction in each type of body-image disturbance. This study was conducted between November 2021 and June 2022 in Sendai, Japan.

We recruited women aged 20–35 years to examine body-image disturbance by community-based sampling because non-clinical individuals can have similar body-image disturbance to patients (3, 4). To reveal neural mechanisms related only to the reduction in body-image disturbance, recruiting non-clinical individuals has advantages. Several studies have investigated brain structures and neural responses while participants doing nothing, and they suggested that people with eating disorders showed neurological differences compared with non-clinical individuals (52–55). These neurological changes are not directly derived from body-image stimuli; thus, it could be related to other factors, such as psychiatric characteristics like depression, anxiety, and so on. In addition, people with anorexia nervosa have serious nutritional problems (2), which also influences brain structure and neural responses (56). Furthermore, understanding the neural mechanisms associated with the reduction in body-image disturbance in non-clinical individuals can facilitate the development of prevention methods, which are particularly important because eating disorders are often refractory to treatment (57, 58). Finally, fMRI studies of non-clinical individuals can define regions of interest (ROIs), which could provide useful information for future neuroimaging studies involving patients. Considering the challenges in recruiting patients with eating disorders, achieving significant results through exploratory voxel-wise analysis is difficult. Therefore, the accumulation of findings from non-clinical individuals is essential for advancing clinical research.

To minimize the recruitment time, individuals older than the legal adult age (i.e., 20 years) in Japan were recruited to this study. The age range of the study participants was similar to that reported in previous studies (46, 47, 49). A previous study investigating body-image-disturbance in women aged 17–40 years found no significant decline with age (59). Therefore, the recruitment of women aged 20–35 years was deemed suitable for including late adolescents. Additionally, the recruitment only of women was appropriate considering the study design, which involved manipulating body width and assumed that the ideal body concept of participants was that a slimmer body is “better”. Despite the diversity in contemporary women’s ideal bodies, including lean, curvy, and toned bodies (60), women’s ideal body image is still less influenced by muscle mass compared to men’s ideal body image (61–64). In particular, Japanese women continue to have slimmer ideal bodies (65).

To examine the degree of body-image disturbance (i.e., perceived–actual and perceived–ideal discrepancies), we used silhouette images distorted in width obtained from each participant’s photograph. Participants estimated their actual and ideal body sizes by examining these distorted silhouettes. Despite the availability of standardized three-dimensional (3D) body images for Japanese women (66), the body mass index (BMI) range of these nine body images did not align with the participants’ BMI. Additionally, previous studies have demonstrated the superiority of using one’s own body instead of standardized images to evaluate body-image disturbance (67–69). Previous fMRI studies investigating body-image disturbance have demonstrated differential neural responses when participants view their own bodies compared to others’ bodies (26, 70). Some behavioral studies have manipulated 3D images of participants’ own bodies (71, 72) or body parts (73) based on changes in actual body shape to increase the realism of stimuli. However, such techniques have been based on body-size databases of White individuals and are applicable only to White participants. Considering a previous study indicating that silhouette images can effectively represent individuals with different body sizes (74), we used participants’ own silhouette images, in line with our previous studies (6, 15). Neural responses during estimation were recorded. Behavioral and neurological data were obtained before and after the interventions.

Participants underwent a brief (1-day) mirror exposure or mental imagery intervention, each lasting 40 min. Brief interventions can effectively reduce scores on questionnaire related to body-image concerns (46, 47). Considering the burden on participants and previous studies, 1-day interventions were implemented in this study. The interventions lasted for 40 min because a previous study showed that at least a 30-min exposure to bodies is needed to reduce body-image concerns.

We conducted multiple regression analysis to investigate neural responses related to the reduction in body-image disturbance. Neural response changes before and after interventions were predicted by changes in perceived–actual or perceived–ideal discrepancy, with controlling for body-image concerns before interventions. In addition to the main analysis, we compared behavioral and neural changes before and after the interventions.

### 2.3 Participants

The study included right-handed women aged 20–35 years with no history of psychiatric disorders or current involvement in weight loss plans. In total, 32 non-clinical women were recruited from the undergraduate or graduate school of Tohoku University (n = 28) or the nearby general population (n = 4). Participants were recruited using advertisements displayed in the university office and a local newspaper. Using the conventional method, we determined the sample size based on similar fMRI studies. Only two previous fMRI studies have compared neural responses before and after interventions targeting body-image disturbance (34, 35); one of them recruited 5 participants (34), and the other recruited 32 people with eating disorders (17 were assigned to a therapy group and 15 to a control group) (35). Thus, we recruited 32 people and assigned them to the mirror-exposure and mental-imagery groups by block randomization, with the block size determined randomly (two, four, or six) (75) and 16 participants assigned to each group.

#### 2.3.1 Pre-screening

The 32 participants underwent a pre-screening test to confirm the presence of body-image disturbance, involving an assessment of perceived–actual and perceived–ideal discrepancies using 3D body images of Japanese women (66). We recruited people with high body dissatisfaction, as the level of body dissatisfaction before the mirror-exposure intervention can influence its effectiveness (46). The perceived–ideal discrepancy was examined because it is correlated with body dissatisfaction (5, 15, 24). Moreover, the perceived–ideal discrepancy was targeted for reduction by the interventions, and this pre-screening process ensured recruitment of individuals with more severe affective disturbance (i.e., those who perceived themselves as overweight compared to their ideal body image). During the pre-screening process, individuals assessed their actual and ideal body sizes based on nine body images with BMI values ranging from 16.5 to 24.5 kg/m^2^. Then, we calculated the “BMI-based actual–ideal discrepancy”. Individuals with perceived–ideal discrepancy ≥ 2 BMI points (i.e., they perceived themselves to be overweight relative to their ideal body by 2 BMI points) were recruited. This criterion was determined based on a previous study, which found that the mean BMI-based actual–ideal discrepancy in Japanese adolescent women was about 2 BMI points (25).

#### 2.3.2 Exclusion criteria

We excluded males as well as individuals who were left-handed, aged< 20 or > 35 years, pregnant, or following weight-loss plans. Individuals with a history of psychiatric disorders, claustrophobia, or metal implants were also excluded from the study. The history of psychiatric disorders was self-reported rather than physician-assessed.

Participants with excessive body movement or an insufficient number of valid scans were excluded (see section 2.7.2 for details). Finally, we analyzed data from 28 participants (14 in each group). A two-sample t-test revealed that there were no significant differences between the groups before the intervention, except for scores on a questionnaire related to body-image concerns (**Table 1**). *P*< 0.05 was considered statistically significant.

**Table 1:** Demographic data of participants and changes in behavioral indices before and after the interventions.

	Mean (SD)				p-value		
	Mirror exposure		Mental imagery		All participants	Group comparison	
	Pre	Post	Pre	Post	Pre vs. Post	Pre	Pre vs. Post
Age	22.1 (1.64)	–	22.8 (2.69)	–	–	0.40	–
BMI (kg/m^2^)	20.9 (3.23)	–	21.5 (2.42)	–	–	0.62	–
Perceived–actual discrepancy (%)	7.33 (4.90)	7.91 (7.61)	6.73 (5.44)	8.88 (5.28)	0.34	0.76	0.65
Perceived–ideal discrepancy (%)	7.99 (7.86)	9.79 (11.6)	8.91 (8.82)	11.5 (10.8)	0.49	0.77	0.89
EDI2	51.4 (13.9)	51.7 (13.2)	64.0 (15.0)	62.1 (14.6)	0.30	0.03*	0.38

The mean age, body mass index (BMI), degree of body-image disturbance, and Eating Disorder Inventory 2 (EDI2) scores of the 28 participants are presented. The degree of body-image disturbance and EDI2 scores were examined before (“Pre” columns) and after (“Post” columns) the interventions. Numbers in parentheses are standard deviation. A two-sample t-test was conducted to examine pre-intervention group differences, and the results are presented as p-values (“Pre” column in the “Group comparison” column). Multiple regression analysis was performed to examine the effects of interventions. The “Pre vs. Post” columns show the p-values of multiple regression analysis to examine the effects of intervention on each discrepancy. The significance of the partial regression coefficient for the main effect of intervention (i.e., data pooled from both groups) is shown in the “All participants” column. The significance of the partial regression coefficient for the interaction term, the interaction between groups (mirror exposure vs. mental imagery) and interventions (pre-intervention vs. post-intervention), is shown in the “Group comparison” column. The threshold for statistical significance was set at p< 0.05.

*p< 0.05.-: Not Applicable.

### 2.4 Experiment outline

**Figure 1:**
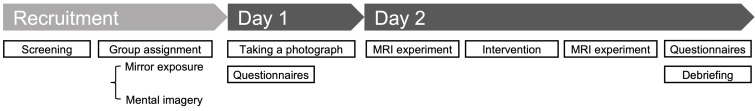
Study outline. Participants presented to the laboratory on 2 days. On Day 1, they were photographed and completed the questionnaires. On Day 2, they completed psychological tasks in an MRI scanner to record their neural responses before and after the interventions. Following completion of the tasks, they filled out the questionnaires again and received a debriefing on the same day. **Figure 2** presents the details of the psychological tasks.

Participants visited our laboratory on 2 days. On day 1, we acquired whole-body photographs of the participants, which were converted into silhouette images. On day 2, the participants completed a psychological experiment in an MRI scanner before and after the intervention (**Figure 1**).

### 2.5 fMRI tasks

#### 2.5.1 Stimuli

To assess each participant’s perceived and ideal body images, we created silhouette images for each participant along with a black square of equal area. The silhouette images were altered in width to represent various body sizes, and the black squares served as a control. This methodology is similar to that used in our previous studies (6, 15). In brief, we obtained photographs of each participant and then converted them into a silhouette image. Both the silhouette images and the black squares were modified in terms of width, consistent with previous studies (3, 15, 74, 76, 77). In total, 21 images were created by varying the width between −25% and +25% of the original width. We altered the width of the original image in increments of 2% from −15% to −1% and from +1% to +15%, resulting in 16 images. Additionally, we altered the original width image in increments of 5% from −25% to −20% and from +20% to +25%, resulting in four images. Therefore, we created a set of 21 images, including the original image (see 6, 15 for further details).

#### 2.5.2 Task design

**Figure 2:**
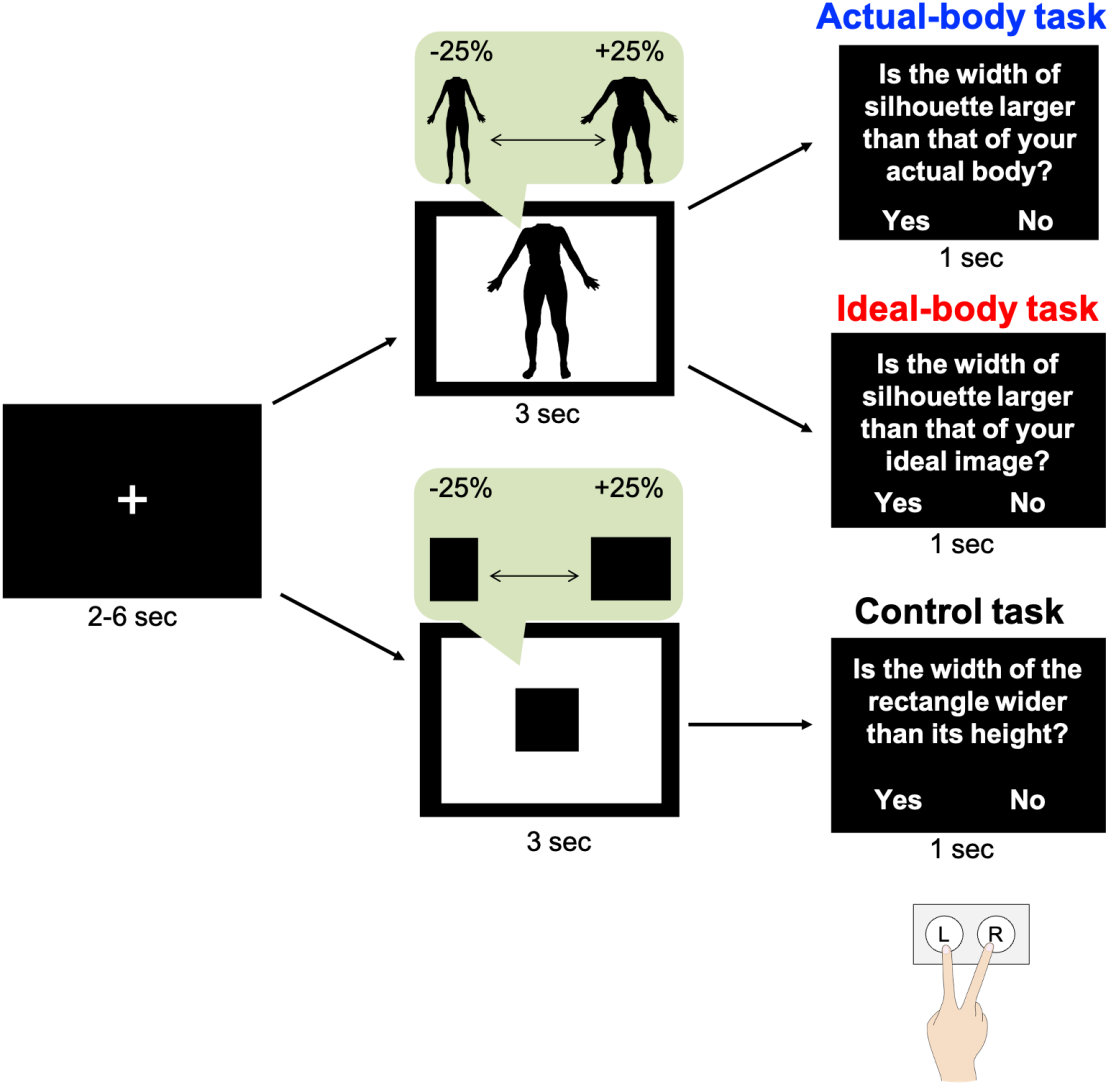
Experiment outline. In each trial, following visual fixation for 2, 4, or 6 s, participants viewed their distorted silhouette image or a black rectangle for 3 s. Subsequently, they responded to questions by pressing the appropriate key. These tasks were similar to those in our previous study (6). The figure has been adapted from the previous study.

During the fMRI task, participants viewed a silhouette image or a black rectangle and then made judgements regarding the width of the image (**Figure 2**). The fMRI task design was similar to that used in our previous study (6), except for the block design and number of trials. Participants completed actual-body, ideal-body, and control tasks in an MRI scanner. Before the task, each participant received a detailed explanation that the stimuli would be distorted in terms of width, and they were required to consider only the width of each image. In the actual-body task, participants judged whether the presented body image was wider than their actual body image, whereas in the ideal-body task, they assessed whether the presented body image was wider than their ideal body image. In the control task, they judged whether the presented black rectangle was wider than a square. This control task was used to isolate brain activity specific to body-image processing. Participants were instructed to make up their mind regarding their answers while viewing the silhouettes or rectangles. Participants practiced the tasks outside the MRI scanner, and they completed 12 trials for each task.

Participants viewed the silhouette image for 3 s and were required to press a button within 1 s. The intervals between trials were set at 2, 4, or 6 s, with a fixation image presented during each interval. Participants viewed their silhouette body image or a black rectangle (21 images with widths from −25% to +25%) twice in random order, resulting in 42 trials for each task. The stimulus presentation was based on a mixed design. Each task was conducted in a separate fMRI session, and participants completed the fMRI tasks in two phases; pre- and post-intervention. Thus, there were three sessions in each phase for a total of six sessions. The order of tasks was counterbalanced across participants. Participants provided their responses by pressing buttons on a keypad held in their right hand, with their index and middle fingers positioned over two buttons representing “yes” and “no.” The correspondence between fingers and responses was counterbalanced across participants. The participants viewed each task through a mirror mounted on the head coil. All tasks were controlled using PsychoPy 2021.1.4 (78).

### 2.6 Intervention tasks

**Figure 3:**
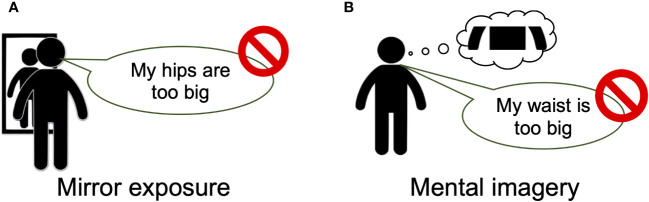
Overview of the interventions. Overview of the mirror-exposure **(A)** and mental-imagery **(B)** interventions. In both interventions, the participants described their body parts without using negative or subjective expressions. Prior to this description, **(A)** participants in the mirror-exposure group were instructed to view the body part, **(B)** whereas those in the mental-imagery group were instructed to imagine the body part. Participants viewed or imagined specific body parts for 10 s, provided two descriptions of each body part in a 25-s period, and then rested for 25 s. This 1-min cycle was repeated for 15 body parts.

An overview of the interventions is provided in **Figure 3**. In both interventions, participants were instructed to describe their body parts without using negative, critical, or subjective expressions after looking at the body part in a mirror (mirror-exposure group) or imagining it (mental-imagery group). In the mirror-exposure group, participants changed into the clothing that they had worn to create the silhouette stimuli and stood in front of a three-way mirror. First, they viewed their bodies freely in the mirror for 3 min to become accustomed to the situation. Next, they focused on a specific body part for 10 s and provided two descriptions of the body part within 25 s, following the experimenter’s instructions (46, 47). The order of body-part description was hair, skin, eyes, nose, mouth, neck, arms, chest, abdomen, waist, buttocks, hips, thighs, calf, and feet (47), with intervals of 25 s between each description. Participants were instructed to imagine that they were providing descriptions for a self-portrait by a blind artist and were instructed to avoid using negative, critical, or subjective expressions when describing their bodies (41). Expressions such as “big,” “fat,” “like,” and “beautiful” were discouraged, and participants were instead encouraged to use descriptions related to color, texture, and proportion. On the other hand, in the mental-imagery group, participants only imagined their body parts, without looking in a mirror, and were given similar instructions to those in the mirror-exposure group, except the instructions to change clothes and look in the mirror (42, 43). Both interventions were conducted by the first author in the same room and took approximately 40 min.

### 2.7 Behavioral data analysis

To investigate the effects of the two interventions on each component of body-image disturbance, we examined the degree of body-image disturbance (see section 2.6.1 for details) and questionnaire scores (see section 2.6.2 for details) before and after each intervention. Statistical analysis was performed to investigate changes in these behavioral indices (see section 2.6.3 for details).

#### 2.7.1 Degree of body-image disturbance

**Figure 4:**
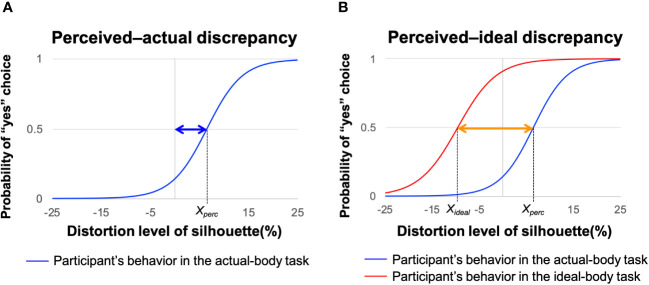
Procedures for estimating perceived–actual and perceived–ideal discrepancies. Representative behavior of a participant during the actual-body (blue line) and ideal-body (red line) tasks based on logistic regression modeling. **(A)** Blue arrow represents the perceived–actual discrepancy, defined as the probability of selecting “yes” on the actual-body task of 0.5 (*X_perc_*). **(B)** Orange arrow represents the perceived–ideal discrepancy, defined as the discrepancy between the probability of selecting “yes” on the actual-body task of 0.5 (*X_perc_*) and the probability of selecting “yes” on the ideal-body task of 0.5 (*X_ideal_*). Adapted from Hamamoto et al. (6).

We determined the perceived–actual and perceived–ideal discrepancies by examining the silhouette sizes of each participant’s perceived-self and ideal images, following the methodology used in previous studies (6, 15). The sizes of the perceived and ideal body images were determined based on participants’ selections in the actual and ideal-body tasks, respectively (**Figure 4**). Logistic regression was used to model participants’ selection behavior as *P_yes_* = 1/[exp(−*α*X − *β*) + 1], where *P_yes_* represents the probability of a “yes” response, *X* indicates the degree of silhouette distortion, and the free parameters *α* and *β* control the slope shape. Notably, the distortion level *X* at which the participants changed their choice from “yes” to “no” corresponds to the size of the perceived or ideal body, denoted as *X_perc_* and *X_ideal_*, respectively.

##### 2.7.1.1 Degree of perceptual disturbance

Perceptual disturbance was defined as the discrepancy between perceived-self body size (*X_perc_* in **Figure 4A**) and actual body size (X = 0 in **Figure 4A**). Thus, the perceived–actual discrepancy was determined as the absolute value of *X_perc_* – zero (blue arrow in **Figure 4A**). For the perceived–actual discrepancy, we followed recent conventions and converted *X_perc_* – zero (blue arrow in **Figure 4A**) to an absolute value (79–81). Although we did not use this convention in our previous studies (6, 15), a reanalysis using absolute values confirmed our previous findings. A greater perceived–actual discrepancy indicated that the participant estimated her body size less accurately, regardless of the direction of misestimation.

##### 2.7.1.2 Degree of affective disturbance

Affective disturbance was defined as the discrepancy between perceived-self body size (*X_perc_* in **Figure 4A**) and ideal body size (*X_ideal_* in **Figure 4B**). Thus, the perceived–ideal discrepancy was obtained by subtracting the participant’s ideal body estimate from her perceived body estimate (*X_perc_* − *X_ideal_*; orange arrow in **Figure 4B**, adapted from 6). A positive perceived–ideal discrepancy value indicated that the participant evaluated her body size as larger than her ideal body.

#### 2.7.2 Questionnaire

To examine participants’ body-image concerns, we used the body-dissatisfaction and drive-for-thinness subscales from the Japanese version of the Eating Disorder Inventory 2 (EDI2) questionnaire (82), which is the most recent Japanese translation. These two subscales are associated with body-image disturbance (83, 84), and scores thereon were obtained before and after the interventions. There were nine and seven items for body dissatisfaction and drive for thinness, respectively. The EDI2 score is typically transformed (e.g., if participants rated an item as 1–3, the score was converted to 0, while score of 4–6 were transformed to 1–3) when evaluating symptom severity in clinical populations. However, we adopted a raw-score rating approach, where we recorded a score of “1” if participants rated an item as “1,” based on a previous study that suggested that this approach is suitable for non-clinical sample (85). We summed the ratings of the 16 questionnaire items. For reference purposes, transformed scores were described in **Supplementary Table 1**. Only one participant showed above a cut-off score for drive for thinness, it has been set at 14< (86, 87). There is no widely used cut-off score for body dissatisfaction in EDI2.

#### 2.7.3 Behavioral changes before and after the interventions

To investigate the effects of mirror exposure and mental imagery, we examined changes in perceived–actual and perceived–ideal discrepancies. Furthermore, we explored the effects of the interventions on eating-disorder characteristics related to body-image disturbance. These behavioral indices were assessed before and after the interventions. Due to significant group differences in the questionnaire scores related to eating-disorder characteristics before the intervention (**Table 1**), we performed analysis of covariance instead of two-way analysis of variance to examine the effect of each intervention while controlling for the pre-existing group difference. The independent variables in the analysis were the main effect of group (mirror exposure = 1, mental imagery = 0), the main effect of intervention (pre-intervention = 0, post-intervention = 1), the interaction between group and intervention, and the confounding factor of questionnaire scores. We evaluated the significance of the main effect of intervention and the interaction term. *P<* 0.05 was considered indicative of statistical significance. The behavioral data were analyzed using R software (version 4.2.1; R Core Team, Vienna, Austria).

### 2.8 fMRI data acquisition and preprocessing

#### 2.8.1 Data acquisition

All images were acquired using a Philips Achieva 3T MRI scanner (Philips, Best, The Netherlands). The whole-brain fMRI dataset was obtained using T2*-weighted gradient echo-planar imaging and comprised 40 gradient-echo images (echo time = 30 ms, flip angle = 85°, slice thickness = 2.5 mm, slice gap = 0.5 mm, field of view = 192 mm, matrix size = 64 × 64). These images covered the entire brain and were obtained using a repetition time of 2,500 ms. In addition, structural whole-brain images were acquired using magnetization-prepared rapid-acquisition gradient-echo, employing the following imaging parameters: repetition time = 6.7 ms; echo time = 3.1 ms; field of view = 192 mm, number of slices = 162, and slice thickness = 1 mm. These parameters were similar to those used in our previous study.

#### 2.8.2 Preprocessing and outlier exclusion

We conducted preprocessing procedures using statistical parametric mapping software (SPM12; Wellcome Center for Human Neuroimaging, London, UK), the CONN functional connectivity toolbox (version 21a; www.nitrc.org/projects/conn, RRID: SCR_009550) (88), and MATLAB software (MathWorks Inc., Natick, MA, USA). We used a preprocessing pipeline implemented in CONN, which included correction for head motion, adjustment of acquisition time across slices, co-registration to anatomical images, spatial normalization using anatomical images and the Montreal Neurological Institute template, and smoothing using a Gaussian kernel with a full width at half maximum of 6 mm.

Participants with outlier values for body movements and the number of valid scans (< [first quartile – 1.5 × interquartile range] or > [third quartile + 1.5 × interquartile range]) were excluded. Two participants were excluded because of excessive body movements (> 4.4 mm), and two were excluded due to an insufficient number of valid scans (< 775 scans).

### 2.9 fMRI data analysis

To investigate the neural-response changes related to reductions in each type of body-image disturbance, we examined the correlation between these changes (i.e., regional neural activity and cross-regional functional connectivity) and the decrease in each type of body-image disturbance (i.e., perceived–actual and perceived–ideal discrepancies). We examined the neural-response changes correlated with reductions in each type of discrepancy at the whole-brain level (see sections 2.8.1.1 and 2.8.1.2 for details). Additionally, we explored neural-response changes in brain regions that we had previously identified as being related to each type of body-image disturbance (6), with a less stringent significance threshold (see sections 2.8.2.1 and 2.8.2.2 for details). We also checked for the same neural responses observed in our previous study before the interventions (see section 2.8.2.3 for details).

#### 2.9.1 Voxel-wise whole-brain analyses

##### 2.9.1.1 Changes in regional neural activity

Data were analyzed within SPM12 (89) using a conventional two-level approach, similar to our previous study (6).

In the first-level analysis of regional neural activity, we aimed to isolate neural responses related to the body width of the presented stimulus for each task. We modelled the event at the onset of the presentation of the silhouette or rectangular image with a duration of 3 s. This conventional canonical neural response was accompanied by a model that parametrically represented modulated neural responses based on the silhouette width in each trial relative to the canonical neural response, consistent with our previous approach (6). Consequently, there were two regressors representing neural responses in each task and the size of the presented stimuli in each phase. The task consisted of three levels: actual body, ideal body, and control. The phase consisted of two levels: pre- and post-intervention. Trials in each task were categorized based on the width of the presented stimulus, because previous studies have suggested that width-dependent neural responses are related to body-image disturbance (6, 90–92). We used two stimulus sizes, as determined previously (6): trials where participants were presented with larger body silhouettes (body width from 0 to +25) and those where they were presented with smaller body silhouettes (body width from −1 to −25). In total, 24 regressors representing neural responses were modelled: two neural-response models × three tasks × two phases × two stimulus sizes. Additionally, we incorporated the six estimated head-movement parameters obtained via preprocessing as regressors (covariates of no interest) to account for the effect of head motion. We applied a high-pass filter with a cut-off of 128 s.

In the second-level analysis of changes in regional neural activity, we performed a multiple regression analysis using contrast images created by subtracting pre-intervention images from post-intervention images i.e., post-intervention (actual-body task − control task) – pre-intervention (actual-body task − control task) and post-intervention (ideal-body task − control task) – pre-intervention (ideal-body task − control task). The contrast related to the actual-body task was used to analyze the reduction in perceived–actual discrepancy, whereas the contrast related to the ideal-body task was used to analyze the reduction in perceived–ideal discrepancy. We assessed the correlations between changes in brain activation and the reduction in each type of discrepancy using multiple regression analysis. The dependent variables were changes in brain activation between pre-intervention and post-intervention (post – pre). The independent variables included the group effect (mirror exposure = 1, mental imagery = 0), changes in the discrepancy (post – pre; perceived–actual or perceived–ideal discrepancy), the interaction between the group effect and changes in the discrepancy, and the confounding factor of the pre-intervention EDI2 score. We examined the significance of the partial regression coefficient of the changes in the discrepancy term to investigate regional neural-activity changes were associated with the reduction in each type of discrepancy. Additionally, we examined the partial regression coefficient of the interaction term to investigate group-specific effects on each type of discrepancy. Uncorrected *p*< 0.001 was considered indicative of statistical significance, corrected for FWE at *p*< 0.05 based on the cluster size.

##### 2.9.1.2 Changes in cross-regional functional connectivity

Data were analyzed using a conventional two-level approach using CONN. Before analyzing cross-regional functional connectivity, we denoised the time-series data derived from the images. Subsequently, we applied band-pass filtering (0.008–0.09 Hz) and removed possible confounding factors, including motion artifacts and white-matter, cerebrospinal-fluid, and task-related effects.

We investigated the functional connectivity from brain regions associated with visual body-image processing, following a similar approach to that of our previous study (6). We created 10-mm spherical seed ROIs for the bilateral extrastriate body area (left: *x*, *y*, *z* = −49, −75, 5; right: *x*, *y*, *z* = 47, −62, 6) and right fusiform body area (*x*, *y*, *z* = 42, −36, −30) based on a previous study that showed involvement of these regions in visual body-image processing (93). These spherical ROIs covered the extrastriate body area and the fusiform body area, both previously identified in fMRI studies focusing on body-image disturbance (18–20, 35).

We conducted a first-level analysis to determine the connectivity between the seed ROIs (i.e., bilateral extrastriate body areas and the right fusiform body area) and whole-brain voxels. Then, in the second-level analysis, we performed multiple regression analysis. The contrasts and regression model used in the second-level analysis were similar to the analyses for regional neural activity. The dependent variables were changes in functional connectivity from the seed ROIs to whole-brain voxels. We assessed the significance of the partial regression coefficients for the changes in the discrepancy and interaction terms. The significance threshold was set at uncorrected *p*< 0.001, corrected for FWE at *p*< 0.05/3 to account for the repetition of tests involving three seed ROIs based on the cluster size.

#### 2.9.2 Analyses of specific brain regions based on our previous findings

##### 2.9.2.1 Changes in regional neural activity

We also performed an ROI analysis based on our previous finding to assess whether the regions previously identified as being associated with each type of body-image disturbance exhibited changes in activity due to the reduction in body-image disturbance. These ROIs, derived from our previous study (6), included the left anterior cingulate cortex (*x, y, z* = −14, 42, 16), which is associated with perceived–actual discrepancy, and the right temporoparietal junction (*x, y, z* = 42, −56, 22), which is associated with perceived–ideal discrepancy.

Next, we adopted a conventional two-level approach, in which the first-level analysis was similar to the voxel-wise analysis. In the second-level analysis, we investigated the correlations between changes in brain activation within these ROIs and the reduction in each type of body-image disturbance using multiple regression analysis, applying the same contrasts and the same model as in the voxel-wise analysis. We assessed the significance of partial regression coefficients for the changes in the discrepancy and interaction terms. Given our *a priori* hypothesis, the threshold for statistical significance was set at *p*< 0.05.

##### 2.9.2.2 Changes in cross-regional functional connectivity

Similar to the analysis of regional neural activity, we conducted ROI analysis (ROI-to-ROI analysis) to test our hypothesis using a less stringent threshold for statistical significance. We examined the functional connectivity of the left extrastriate body area with target ROIs, including the left anterior insula (*x, y, z* = −40, −2, 14) and right precuneus (*x, y, z* = 6, −72, 40). We observed that the functional connectivity of the left extrastriate body area was correlated with the perceived–actual discrepancy in the left anterior insula and the perceived–ideal discrepancy in the right precuneus.

Similar to the voxel-wise functional connectivity analysis, denoising was performed before the first- and second-level analyses. The first-level analysis was performed to determine the connectivity between the left extrastriate body area and target ROIs. The second-level analysis was similar to the whole-brain analysis. Furthermore, the significance of partial regression coefficients for changes in the discrepancy and interaction terms was examined. *P*< 0.05 was considered indicative of statistical significance.

##### 2.9.2.3 Verification of previous findings

We confirmed our previous findings (6) using the pre-intervention fMRI data obtained in the present study.

In the second-level analysis of regional neural activity, we confirmed the correlations of brain activation with the perceived–actual and perceived–ideal discrepancies observed in our previous study (6). Multiple regression analysis was performed using pre- and post-intervention contrast images; (pre-intervention [actual-body task − control task]) and (pre [ideal-body task − control task]). The dependent variables were brain activation in ROIs, and the independent variables were perceived–actual discrepancy, perceived–ideal discrepancy, participant BMI, and the slope of the logistic curve for the actual- or ideal-body task. Non-target regressors (e.g., perceived–ideal discrepancy was the confounding factor when analyzing the neural response associated with perceived–actual discrepancy), BMI, and the slope of the logistic curve were modelled separately as confounding factors. The threshold of statistical significance was set at *p*< 0.05. The models used for multiple regression analysis were similar to those used in our previous study (6).

The first-level analysis of functional connectivity was performed to determine the connectivity between the seed ROI (i.e., the left extrastriate body area) and the target ROIs (i.e., the left anterior insula and right precuneus). In the second-level analysis, we performed multiple regression analysis using the same contrasts as in the analysis of regional neural activity. The dependent variables were functional connectivity between the seed ROI and targeted ROIs, and the independent variables were perceived–actual discrepancy, perceived–ideal discrepancy, and participant BMI. Non-targeted regressors of the discrepancy (e.g., perceived–ideal discrepancy was the confounding factor when analyzing the neural response associated with the perceived–actual discrepancy) and BMI were modelled separately as confounding factors. Unlike the analysis of regional neural activity, the logistic curve slope was not included in the model because we did not categorize data according to the silhouette size in the functional connectivity analysis. These methodologies were similar to those used in our previous study (6). The threshold of statistical significance was set at *p*< 0.05.

## 3 Results

### 3.1 Behavioral data

**Figure 5:**
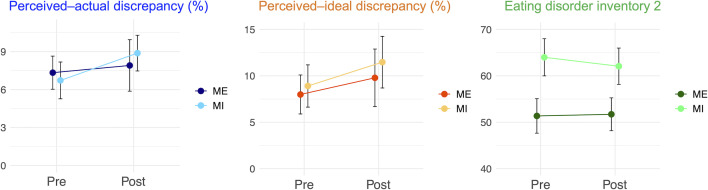
Behavioral changes before and after the interventions. Behavioral changes before and after the interventions: perceived–actual discrepancy, perceived–ideal discrepancy, and Eating Disorder Inventory 2 scores are presented. Error bars represent standard error. ME, mirror exposure; MI, mental imagery.

**Table 1** and **Figure 5** present the pre- and post-intervention changes in behavioral indices. Body-image disturbance was not significantly reduced. The main effects of interventions were not significant (perceived–actual discrepancy: $\eta_{p}^{2}$ [partial *η^2^*] = 0.02, *p* = 0.36; perceived–ideal discrepancy: $\eta_{p}^{2}=0.01,p=\;0.40$ , EDI2: $\eta_{p}^{2}=0.001,p=\;0.54$ ), implying that there were no significant “group-common” intervention effects on any behavioral indices. Similarly, the interactions between group and interventions were not significant (perceived–actual discrepancy: $\eta_{p}^{2}=0.004,p=\;0.65$ ; perceived–ideal discrepancy: $\eta_{p}^{2}=0.0004,p=\;0.89$ ; EDI2: $\eta_{p}^{2}=0.002,p=\;0.38$ ), implying that there were no significant group differences in terms of the effects on behavioral indices.

### 3.2 Voxel-wise whole-brain analysis

#### 3.2.1 Changes in regional neural activity

**Figure 6:**
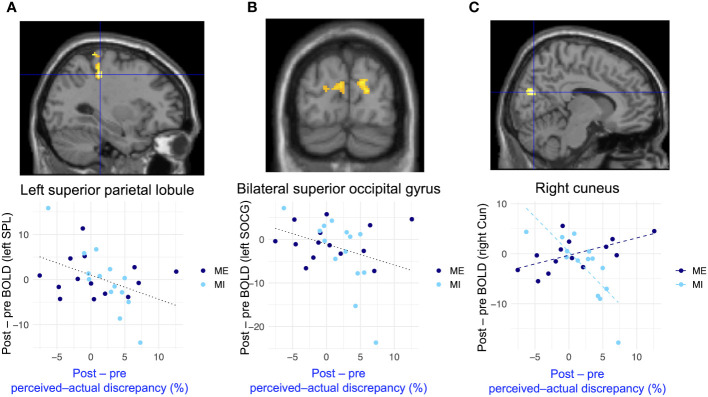
Group-common and group-specific neural responses associated with reduced perceived–actual discrepancy detected by whole-brain analysis. **(A, B)** present group-common changes in brain activation associated with reduced perceived–actual discrepancy during the actual-body task. **(C)** shows the group-specific changes in brain activation associated with reduced perceived–actual discrepancy during the actual-body task. There were significant differences between groups regarding changes in brain activation associated with reduced perceived–actual discrepancy. Black dotted lines in the bottom row represent the regression lines for both groups, whereas dashed lines indicate the regression coefficient for each group. BOLD, blood oxygenation level-dependent; SPL, superior parietal lobule; SOCG, superior occipital gyrus; Cun = cuneus.

**Figure 6** and **Table 2** present the results of the voxel-based whole-brain analysis of regional neural activity. The analysis revealed a significant group-common effect regarding individual differences in reductions in perceived–actual discrepancy. The post–pre change in the perceived–actual discrepancy was negatively correlated with post−pre activation changes in the left superior parietal lobule and bilateral occipital gyri, irrespective of the intervention group (**Figures 6A, B**, **Table 2**). Furthermore, there was a negative correlation between the post−pre change in perceived–actual discrepancy and post−pre activation changes in the right cuneus in the mental-imagery group (**Figure 6C**, **Table 2**). Participants who experienced a reduction in perceived–actual discrepancy also exhibited increased activation in the left superior parietal lobule and bilateral superior occipital gyri, irrespective of the intervention group, whereas the increase in activation in the right cuneus was observed only in the mental-imagery group. Notably, there were no significant group-common or group-specific neural responses related to the post−pre change in perceived–ideal discrepancy.

**Table 2:** Activated brain regions detected by exploratory whole-brain analysis.

Anatomical label	MNI coordinates (peak)				t-value	Cluster	
	L/R	x	y	z		Size (voxels)	Corrected p-value
Changes in regional neural activity							
Contrast: actual-body task > control task							
Common neural response							
Superior parietal lobule	L	-30	-32	46	6.63	268	*p* = 0.001
Superior occipital gyrus	L	-20	-88	24	4.43	153	*p* = 0.02
	R	20	-82	16	4.71	123	*p* = 0.04
Group specific neural response							
Cuneus	R	10	-74	22	5.38	129	*p* = 0.04
Changes in cross-regional functional connectivity							
Contrast: ideal-body task > control task							
Group specific neural response							
Precuneus (from left extrastriate body area)	R	4	-52	56		111	*p* = 0.004

Activation peaks showed significant correlations between reductions in each body-image disturbance and changes in neural responses. There was a main effect of a decrease in perceived–actual discrepancy (group-common changes in regional neural activity) and the interaction between groups (mirror-exposure group vs. mental-imagery group) and the decrease in perceived–actual discrepancy (group-specific changes in regional neural activity). There was a significant interaction between group and the decrease in perceived–ideal discrepancy (group-specific changes in cross-regional functional connectivity). The details of each activation peak are provided, including the MNI coordinates (x, y, z), t-value, cluster size (voxel size = 2 × 2 × 2 mm^3^), and corrected p-value. The contrasts were obtained by analyzing the differences between parametrically modulated regressors. The threshold for statistical significance was set at uncorrected p< 0.001, with correction for FWE at p< 0.05 based on the cluster size. L and R indicate the left (L) and right (R) hemispheres, respectively.

MNI, Montreal Neurological Institute.

#### 3.2.2 Changes in cross-regional functional connectivity

**Figure 7:**
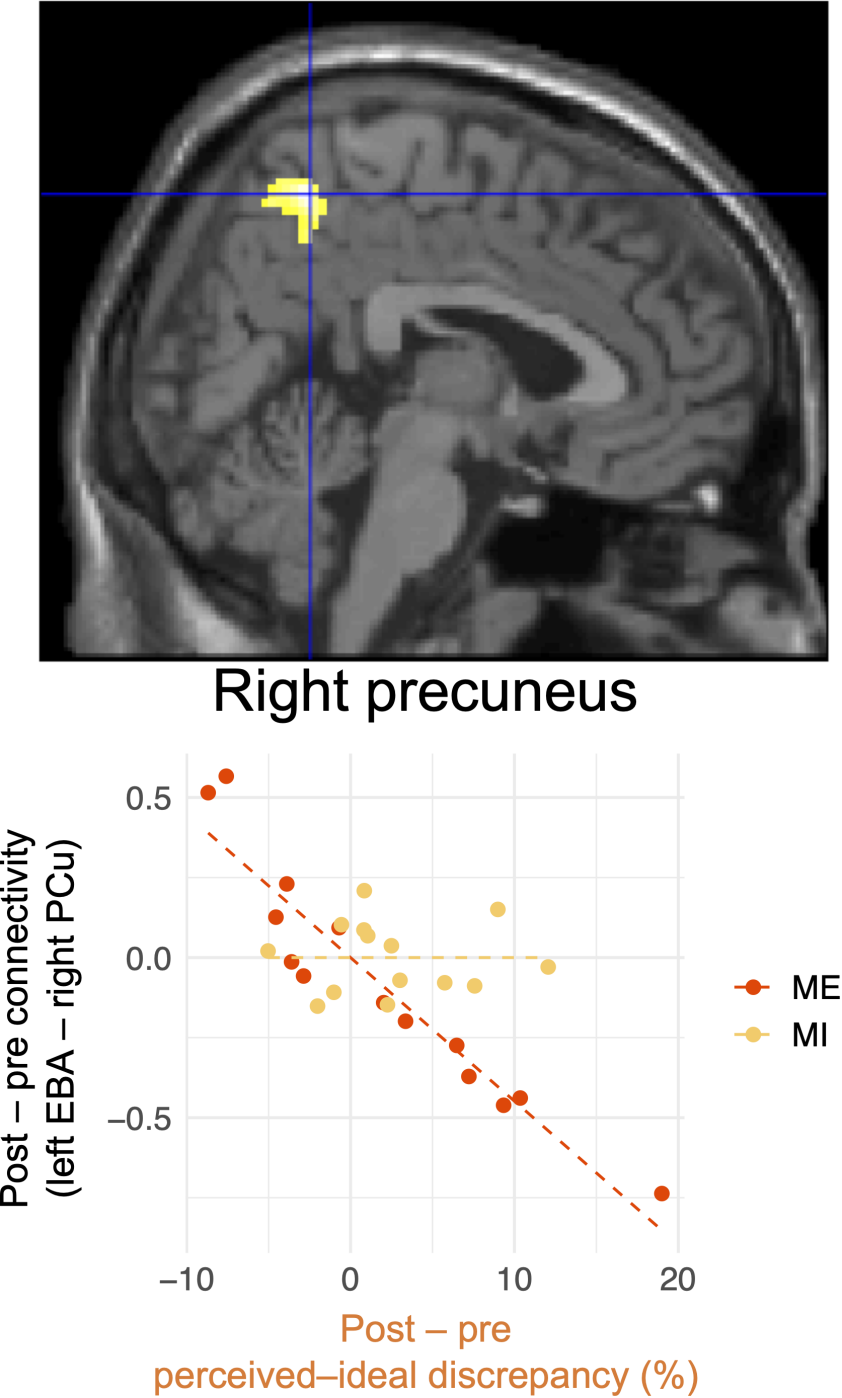
Changes in functional connectivity associated with reduced perceived–ideal discrepancy detected by whole-brain analysis. Reduced functional connectivity between the left extrastriate body area and the right precuneus was associated with a decrease in perceived–ideal discrepancy during the ideal-body task in the mirror-exposure group. There were significant differences between groups in terms of correlations between changes in functional connectivity and perceived–ideal discrepancy. EBA, extrastriate body area; PCu, precuneus.

**Figure 7** and **Table 2** present the results of voxel-based whole-brain functional connectivity analysis. A group-specific effect related to reduction in perceived–ideal discrepancy was observed as a change in functional connectivity from the left extrastriate body area to the right precuneus. A negative correlation was seen in the mirror-exposure group (k = 111, *p* = 0.001; **Figure 7**, **Table 2**), indicating that participants in the mirror-exposure group who experienced a reduction in perceived–ideal discrepancy also exhibited an increase in functional connectivity from the left extrastriate body area to the right precuneus. The right precuneus identified in this study did not overlap with the right precuneus identified in our previous study (6). There were no significant group-common effects in the correlations between reduction in body-image disturbance and changes in functional connectivity from the left extrastriate body area. Additionally, there were no significant group-specific changes in functional connectivity related to the decrease in perceived–actual discrepancy.

### 3.3 Analyses of brain regions identified in previous studies

#### 3.3.1 Changes in regional neural activity

**Figure 8:**
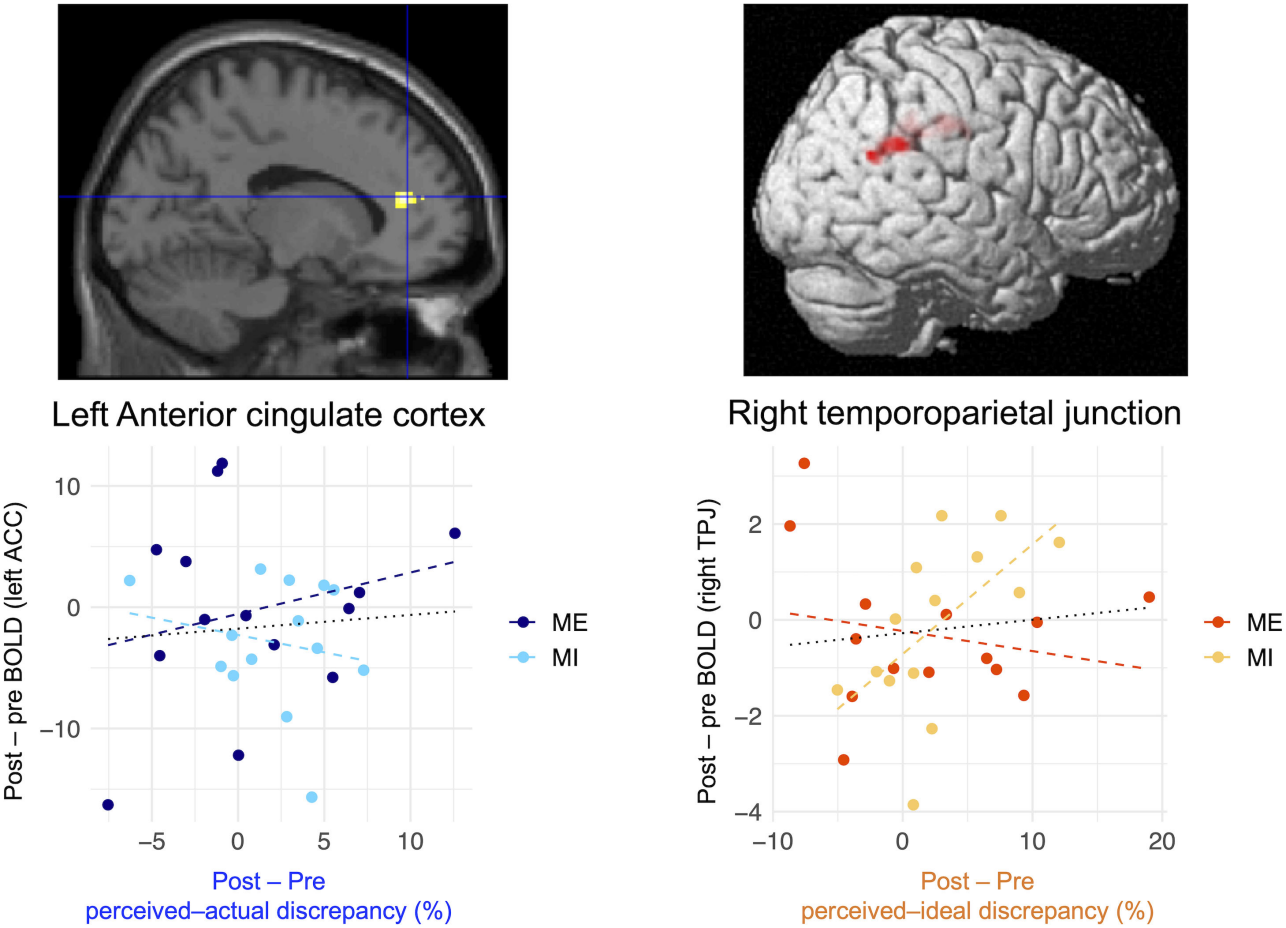
Correlation between changes in brain activation in the ROIs and the reduction in each body-image disturbance. Multiple regression analysis demonstrated that changes in brain activation in the right temporoparietal junction were positively correlated with a reduction in perceived–ideal discrepancy in both groups; this effect was significantly stronger in the mental-imagery group. Conversely, there were no significant associations between changes in brain activation and reduction in perceived–actual discrepancy. Scatter plots were created to visualize the results. Black dotted lines in the bottom row represent the regression lines for both groups, whereas dashed lines represent the regression coefficients for each group. Brain images were adapted from a previous study (6). BOLD, blood oxygenation level-dependent; ACC, anterior cingulate cortex; TPJ, temporoparietal junction.

The results of the ROI analysis of regional neural activity are presented in **Figure 8**. There were significant group-common and group-specific effects on the changes in brain activation in the right temporoparietal junction. The post−pre decrease in perceived–ideal discrepancy was positively correlated with the post−pre activation changes in the right temporoparietal junction (*β* = 0.25, *p* = 0.02; **Figure 8**), and this positive correlation was significantly stronger in the mental-imagery group compared to the mirror-exposure group (*β* = −0.30, *p* = 0.01; **Figure 8**). Participants in the mental-imagery group who experienced a reduction in perceived–ideal discrepancy also exhibited deactivation in the right temporoparietal junction. Conversely, no significant group-common (*β* = −0.15, *p* = 0.78; **Figure 8**) or group-specific (*β* = 0.54, *p* = 0.40; **Figure 8**) effects were observed in the change in brain activation in the left anterior insula.

#### 3.3.2 Changes in cross-regional functional connectivity

The ROI analysis of changes in functional connectivity revealed that there were no significant group-common or group-specific effects. The group-common effects on correlations between the decrease in body-image disturbance and changes in functional connectivity from the left extrastriate body area to target ROIs were not significant (left anterior insula, *β* = 0, *p* = 0.88; right precuneus, *β* = −0.01, *p* = 0.79). Similarly, there were no significant group-specific effects on correlations between the reduction in body-image disturbance and changes in functional connectivity from the left extrastriate body area to target ROIs (left anterior insula, *β* = 0.01, *p* = 0.74; right precuneus, *β* = 0.01, *p* = 0.72).

#### 3.3.3 Verification of previous findings

The correlation between perceived–actual discrepancy and left anterior insula was marginally significant (*β* = 0.41, *p* = 0.08), whereas that between perceived–ideal discrepancy and right temporoparietal junction was not significant (*β* = 0.01, *p* = 0.78).

We failed to replicate our previous findings in the network-level analysis. With regard to pre-intervention contrast images and pre-intervention perceived–actual and perceived–ideal discrepancies, the ROI-to-ROI analysis revealed no significant correlation between perceived–actual discrepancy and functional connectivity from the left extrastriate body area to the left anterior insula (*β* = 0.01, *p* = 0.33). There were no significant correlations between perceived–ideal discrepancy and functional connectivity from the left extrastriate body area to the right precuneus (*β* = 0.01, *p* = 0.25).

## 4 Discussion

We investigated the neural mechanisms underlying the reduction in perceptual and affective disturbances. To reveal these mechanisms, we compared the behavioral and neural indices of non-clinical young women before and after mirror-exposure and mental-imagery interventions. There were no significant behavioral changes after the interventions; however, we identified neural responses related to individual differences in reductions in each type of body-image disturbance. A reduction in perceptual disturbance was associated with increased activation in bodily visuospatial processing regions, such as the left superior parietal lobule, bilateral superior occipital gyri, and right cuneus. On the other hand, a reduction in affective disturbance was related to changes in neural responses in socio-cognitive processing regions, such as decreased activation in the right temporoparietal junction and increased functional connectivity from the left extrastriate body area to the right precuneus. In this study, we tested our hypothesis that changes in attentional and socio-cognitive processing are associated with a reduction in perceptual and affective disturbances, respectively. However, our results provide evidence only for a relationship between socio-cognitive processing changes and a reduction in affective disturbance. Considering the small sample size of the study and the partial replication of our previous findings, our results should be considered preliminary.

### 4.1 Lack of behavioral improvements after interventions targeting body-image disturbance

There are two possible reasons for the lack of significant behavioral improvements after the interventions. First, the extent of body-image disturbance and body dissatisfaction among the participants might have been insufficient. Previous studies have shown that individuals with stronger body dissatisfaction exhibit greater improvements following interventions (46); thus, participants with less body-image disturbance and body dissatisfaction would exhibit smaller improvements. To select participants with significant body-image disturbance and body dissatisfaction, we recruited individuals with BMI-based perceived–ideal discrepancy values > 2. However, there were no significant correlations between BMI-based perceived–ideal discrepancy in the pre-screening test and silhouette-based perceived–ideal discrepancy in the main experiment (r = −0.02, *p* = 0.90). As a result, the perceived–ideal discrepancy was similar to our previous studies (6, 15), implying that we failed to recruit individuals with greater body-image disturbance. The lack of a significant correlation between BMI-based perceived–ideal discrepancy and silhouette-based perceived–ideal discrepancy may be attributable to the BMI range in the pre-screening test. The BMI range covered by the 3D images was 16.5–24.5 kg/m^2^ (66), but three study participants had a BMI outside of this range. Moreover, eight participants selected the largest body as their own body or the smallest body as the ideal body, implying that these 3D images did not accurately reflect participants’ perceived and ideal images. Therefore, future studies should conduct pre-screening tests using the same task as the one used in the main experiment of this study.

Second, it is possible that the intervention was not administered for a sufficiently long duration. We selected the intervention duration based on a previous study that demonstrated that body dissatisfaction was reduced after 30-min exposure to one’s own body (43). Considering cultural differences in body images, differences may also exist in the time required to reduce body dissatisfaction (94, 95). It is possible that participants felt greater uneasiness regarding their body during the interventions (96). Additionally, 1-day interventions may not be sufficiently long to reduce body-image disturbance. We conducted 1-day interventions to reduce body-image disturbance based on previous studies that demonstrated reduced questionnaire scores regarding body-image concerns (46, 47). However, several studies administered relatively long interventions lasting for several weeks (41–43). Thus, it is possible that the effects of our interventions were weakened by their short duration and lack of repetition. To examine these factors, further studies are needed to determine the effects of 1-day interventions with longer duration as well as the influence of body uneasiness.

### 4.2 Changes in neural responses related to reduction in perceptual disturbance

Our results imply that increased activation in the visuospatial processing regions when estimating one’s body size is associated with a reduction in perceptual disturbance. We found that increased activation in the left superior parietal lobule, bilateral superior occipital gyri, and right cuneus was associated with a decrease in perceived–actual discrepancy during the actual-body task. Notably, there was significantly increased activation in the right cuneus in the mental-imagery group. The left superior parietal lobule is involved in bodily visuospatial processing (97), and people with eating disorders exhibit less brain activation in the left superior parietal lobule when viewing their own body (98). The superior occipital gyrus and right cuneus are also involved in bodily visuospatial processing (99–102). In line with the previous finding that brain activation in visual-processing regions declines in people with eating disorders (3, 18–20), we found a significant inverse relationship between perceptual disturbance and brain activation in regions involved in bodily visuospatial processing. These findings imply that perceived–actual discrepancy is decreased due to greater visuospatial processing when estimating one’s body size. These group-common relationships between changes in brain activation in regions involved in bodily visuospatial processing and the decrease in perceived–actual discrepancy are attributable to the use of objective expressions rather than avoiding negative expressions. When participants in both interventions described the shape of their body parts, they were encouraged to use objective expressions, such as describing proportion and length. Participants who experienced a decrease in perceived–actual discrepancy likely considered their body image objectively during the actual-body task, which may have been reflected in increased activation in bodily visuospatial processing regions.

The group-specific neural responses seen in this study imply that mental imagery is more effective than mirror exposure in reducing perceptual disturbance. As demonstrated in **Figure 6C**, brain activation in the right cuneus increased with a decrease in perceived–actual discrepancy in the mental-imagery group. Moreover, as shown in **Figures 6A, B**, group-common neural responses related to the reduction in perceived–actual discrepancy were mainly observed in participants in the mental-imagery group. Although the interaction did not reach statistical significance, the neural responses in the left superior parietal lobule and bilateral superior occipital gyri may also be associated with the effect of mental imagery rather than representing group-common effects, similar to the neural response in the right cuneus. With regard to the acute effects of single-session interventions, it is suggested that mental imagery contributes to reduced perceptual disturbance and enhancement of visual processing. During mental imagery, participants imagined a specific body part and considered how to describe it. Thus, participants had to recollect their body parts, leaving limited time and cognitive resources for negative feelings toward these body parts. Additionally, participants in the mental-imagery group may have imagined their body part from both a first-person and mirror-like point of view, potentially enhancing their real-time perception-driven inputs when estimating their body sizes rather than relying on attitudes and beliefs related to their bodies (103). The specific improvements in bodily visual processing were mainly derived from mental imagery, implying that interventions can be conducted with a relatively low risk of side effects to prevent misestimation of one’s body size. Mental imagery would be less harmful because, compared to exposure therapies, it is less likely to increase body-image concerns (20, 43, 49). Additionally, mental imagery does not require special tools, such as a full-length mirror, and paradigms can be conducted online. Therefore, future research investigating the relationship between perceptual disturbance and mental imagery can be conducted online, enabling the recruitment of more participants from a wider population.

The brain regions related to the reduction in perceptual disturbance imply that perceptual disturbance is not caused solely by visual processing problems, as relatively higher visual processing is related to a reduction in perceptual disturbance. We observed involvement of the left superior parietal lobule, bilateral superior occipital gyri, and right cuneus. Our results are consistent with the conventional hypothesis that perceptual disturbance is related to visual processing areas, such as the extrastriate body area, fusiform body area, and parietal lobule (3, 18–20, 104). The brain regions related to the reduction in perceptual disturbance are also involved in middle- to higher-order visual processing, including multisensory integration (105–108). We extracted neural responses specific to body image by subtracting the actual-body and control task data; thus, the neural response included recognizing that the presented stimulus was one’s own body. Therefore, certain higher cognitive functions specific to one’s own body, such as multisensory integration, are associated with a reduction in perceptual disturbance rather than bottom-up solely visual processing changes. This observation is consistent with our previous assumption that neural mechanisms underlying perceptual disturbance are not solely reliant on simple visual processing (6).

### 4.3 Changes in neural responses related to reduction in affective disturbance

With regard to neural responses related to reduction in affective disturbance, both regional neural activity and cross-regional functional connectivity imply the involvement of socio-cognitive processing. ROI analysis revealed that the brain activation in the right temporoparietal junction decreased with a reduction in perceived–ideal discrepancy, consistent with our previous finding that the right temporoparietal junction was positively correlated with perceived–ideal discrepancy (6). Additionally, whole-brain functional connectivity analysis demonstrated that the functional connectivity between the left extrastriate body area and the right precuneus increased with a reduction in perceived–ideal discrepancy. This finding is also consistent with our previous result that functional connectivity from the left extrastriate body area to the right precuneus was negatively correlated with perceived–ideal discrepancy. The right temporoparietal junction and right precuneus are associated with socio-cognitive processing, such as thinking about others’ thoughts (30, 31, 109–111). The similar results between our present and previous studies provide additional evidence of the neural relationship between affective disturbance and socio-cognitive processing, which is conceptually consistent with the widely accepted model of the development of body-image disturbance (32).

Our findings of group-specific neural responses imply that both interventions were effective in reducing affective disturbance, although different mechanisms might be involved in each intervention. Despite the significant group effects, **Figure 4B** demonstrates that the relationship between deactivation in the right temporoparietal junction and the reduction in perceived–ideal discrepancy was stronger in the mental-imagery group. On the other hand, the correlation between the reduction in perceived–ideal discrepancy and changes in functional connectivity from the left extrastriate body area to the right precuneus were stronger in the mirror-exposure group. We previously inferred that higher activation in the right temporoparietal junction and lower functional connectivity from the left extrastriate body area to the precuneus were associated with thoughts about ideal body images prevalent in society. However, this study demonstrated that these neural responses were influenced by different interventions, implying that each neural response reflects distinct psychological processes. Regarding the differences between mirror exposure and mental imagery, mirror exposure is a more visually focused intervention. It is possible that reduced visual input related to one’s body during mental imagery allowed the participants to concentrate on objectively evaluating their bodies without any increase in body dissatisfaction. Conversely, mirror exposure may reduce body-image concerns, as suggested by a previous report that mirror exposure reduces the frequency of negative thoughts and feelings of ugliness compared to mental imagery (42). Future studies should evaluate other indices of body-image concerns as well as perceived–ideal discrepancy.

Our findings that the changes in the right temporoparietal junction and functional connectivity between the left extrastriate body area and the right precuneus varied between the different interventions imply distinct roles of these regions in body-image processing. Based on previous studies, we hypothesized that the precuneus is closely related to body-specific socio-cognitive processing. Both the temporoparietal junction and precuneus are related to socio-cognitive processing, including understanding others’ thoughts (30, 109–111); however, only the precuneus participates in contemplation of others’ evaluations and descriptions regarding the appearance of one’s own body (31). A previous study compared neural responses when participants evaluated their own body parts (e.g., “I think my arms are bony”) and when they surmised evaluations of their body parts by their friends (e.g., “My friend thinks my neck is slender”). Therefore, the decreased activation in the right temporoparietal junction in the mental-imagery group may indicate that participants stopped contemplating vague evaluations from others, such as “beautiful”, “ideal”, or “unpreferable”. On the other hand, the increase in functional connectivity between the left extrastriate body area and the right precuneus in the mirror-exposure group indicates that participants stopped thinking about the evaluations of others regarding specific body parts. In line with this, a previous study demonstrated that mirror exposure reduces the frequency of negative thoughts and feelings of ugliness compared to mental imagery (42). Considering that both the temporoparietal junction and precuneus are related to the expression of affective disturbance (6), neural response changes in both regions could be equally important. Therefore, to address both processing aspects, conducting both mirror exposure and mental imagery could be effective. A previous study conducted mirror exposure in a laboratory with an experimenter, and participants were asked to engage in mental imagery at home (43). Such an approach may be the most effective for reducing the affective component.

### 4.4 Academic and clinical contributions

Our results reveal both differences and similarities between the neural mechanisms associated with a reduction in body-image disturbance and the degree of body-image disturbance. In our previous study, we found a positive correlation between activation in the left anterior cingulate cortex and perceived–actual discrepancy (6), which was replicated in the present study. However, in this study, we did not observe significant deactivation in this region accompanied by reduction in perceived–actual discrepancy. Instead, we identified increased activation in regions associated with bodily visuospatial processing. This inconsistency implies that the neural mechanisms related to the expression of perceptual disturbance differ from those related to its reduction. Attentional processing may influence the degree of perceptual disturbance, whereas bodily visuospatial processing could be related to its reduction. This challenges the assumption that perceptual disturbance is related solely to bodily visuospatial processing, which has been made in several fMRI studies that compared people with eating disorders and healthy individuals (3, 20, 26–28). These studies have revealed differences in neural responses in bodily visuospatial processing regions between people with eating disorders and healthy individuals. Our results imply that the neural responses observed in previous studies represent development or reduction of perceptual disturbance rather than neural responses related to the degree of perceptual disturbance.

Unlike perceptual disturbance, expression and reduction of affective disturbance share a common neural mechanism. We observed changes in neural responses associated with a reduction in perceived–ideal discrepancy in brain regions where we had previously identified neural responses that correlated with perceived–ideal discrepancy (6). In particular, we observed that reduced activation in the right temporoparietal junction, and increased functional connectivity between the left extrastriate body area and the right precuneus, were accompanied by a decrease in perceived–ideal discrepancy. These results imply that socio-cognitive processing is related to both expression and reduction of affective disturbance, implying that changes in affective disturbance are intertwined with socio-cognitive processing. Therefore, our results imply that perceptual and affective disturbances develop, persist, and change independently. These results also provide information useful for the development of biomarkers for the prevention and treatment of each component of body-image disturbance, as well as novel interventions that directly manipulate neural responses. Moreover, our findings imply that, as observed previously, the neural mechanisms underlying the expression of each type of body-image disturbance are relatively robust, as evidenced by the replication of the results regarding the left anterior cingulate cortex and the neural mechanisms underlying the reduction in affective disturbance.

Another clinical implication of our study is the potential for improving intervention instructions. We did not observe significant behavioral improvements related to body-image concerns. One common aspect of the two interventions was the instruction to avoid negative expressions when describing one’s body. Consistent with previous studies (41, 46), we encouraged participants to avoid using subjective expressions, including both positive (e.g., “beautiful” and “like”) and negative expressions. Instead, participants were encouraged to use objective neutral expressions related to length, color, and ratio. However, our non-significant results imply that, instead of encouraging the use of objective neutral expressions, positive expressions should be encouraged, although they are subjective. Some participants described the size and length of their body parts in comparison to other objects, which could increase body-image concerns despite the lack of use of negative expressions. Moreover, it has been reported that the use of positive subjective expression when describing one’s body is associated with reduced body-image concerns (43, 48, 49). Therefore, mirror exposure and mental imagery would be improved by instructing participants to avoid subjective negative expressions and encourage positive ones.

### 4.5 Limitations

This study had several limitations. First, we could only partially replicate the findings of our previous study, which investigated the neural responses associated with each type of body-image disturbance. We did not observe a positive correlation between perceived–actual discrepancy and functional connectivity from the left extrastriate body area to the left anterior insula, a positive correlation between perceived–ideal discrepancy and the right temporoparietal junction, or a negative correlation between perceived–ideal discrepancy and functional connectivity from the left extrastriate body area to the right precuneus. The most likely reason for our inability to replicate these results is the limited sample size. In our previous study, we analyzed data from 36 women, whereas in the current study, we analyzed data from only 28 women. However, we do not think that the previous findings were obtained by chance, as some of them were indirectly supported in the current study. This study showed reduced activation in the right temporoparietal junction in association with decreased perceived–ideal discrepancy in the mental-imagery group (**Figure 4B**). Additionally, the mirror-exposure group demonstrated increased functional connectivity from the left extrastriate body area to the right precuneus, associated with a decrease in perceived–ideal discrepancy (**Figure 7**). Although a replication study with a similar sample size to our previous study is warranted, the present results should be considered consistent with those of the previous study.

Second, the lack of significant collective behavioral improvements warrants discussion, even though our primary objective concerning neural mechanisms was achieved through individual analysis. We have considered two possible explanations: 40-min interventions were not sufficiently long to reduce body-image disturbance for Japanese individuals, or 1-day interventions were not sufficiently effective. Brief interventions are highly valuable considering the worldwide shortage of mental health workers (112, 113), particularly in situations such as pandemics where in-person visits are limited. Therefore, it is crucial to determine whether brief interventions can reduce body-image disturbance without the need for repeat visits over several weeks. In future studies, the duration of interventions should be extended, and their effects should be examined in a larger population. Comparison with long-term interventions spanning several weeks would provide useful information. Additionally, this study investigated only the acute effects of interventions; therefore, the long-term effects of interventions should be investigated in future studies.

Third, our sample size was small. We analyzed 36 participants in our previous fMRI study evaluating individual differences (6), whereas this study enrolled only 28 participants. Although our sample size is not excessively small compared to recent fMRI studies investigating individual differences (114, 115), it was not sufficiently large to ensure replicability (116). However, we believe that our results were relatively robust because they are in line with those of our previous study (6) and other studies (3, 18–20). The small sample size may have contributed to the non-significant behavioral improvements. In the present study, the main effect of intervention on perceived–actual and perceived–ideal discrepancies was small according to conventional effect size classifications (small, 0.01–0.05; medium, 0.06–0.13; large, > 0.14) (117). The largest effect size was *η_p_^2 ^* = 0.02. We calculated the number of participants required for adequate power based on the effect size from ANCOVA using G*Power 3.1 (118). Power analysis using G*Power with *η_p_^2 ^* = 0.02 (the largest effect size for the main effect of intervention on the perceived–actual discrepancy), α = 0.05, and power = 0.8 demonstrated that 387 participants would be needed. Considering that previous studies involving short-term interventions reported significant results with< 100 participants, our study may not have appropriately estimated the effect size of the interventions. Furthermore, *post-hoc* power analysis for the analysis of covariance using G*Power 3.1 (118) based on the main effect of intervention revealed a power of 0.11 for perceived–actual discrepancy, 0.08 for perceived–ideal discrepancy, and 0.05 for the EDI2 score. Based on the interaction effect, the power was 0.06 for perceived–actual discrepancy, 0.05 for perceived–ideal discrepancy, and 0.06 for the EDI2 score. These results also imply that our sample size was not sufficiently large to accurately estimate effect sizes. Thus, in both the neurological and behavioral context, future studies with larger sample sizes are needed.

Fourth, the age range was another study limitation. Our results are probably applicable to late adolescents (aged around 18 years), as suggested by a previous study that found that body-image disturbance in women aged 17–40 years did not decline with age (59). However, early and middle adolescents are the most likely to develop eating disorders (2). Additionally, there is growing interest in exploring body-image disturbance in other groups, such as men and transgender individuals (62, 119). Future research should involve more diverse groups, including patients, men, those with other gender identities, and early to middle adolescents as well as people with eating disorders.

Fifth, we did not control for education level, which influences body perception and body dissatisfaction (120, 121). Even though only four participants were from outside the university, it could influence our results considering our small sample size. Nevertheless, this would affect the generalizability of our results. To enhance generalizability, future studies should control for the socioeconomic status and education level of participants recruited from the general population.

Finally, our stimuli could not investigate changes in participants’ estimates of their body circumference, tone, and curviness. A previous study demonstrated that virtual-reality-based interventions improved the ability to estimate one’s body circumference (122). Moreover, the ideal body image is diverse, including toned and curvy bodies (60). However, the silhouette image used in the present study can be employed only to estimate body width; 3D body images are needed to examine the ability to estimate body size, including circumference, muscle, and curviness, in an MRI scanner. Future studies should use similar stimuli to investigate the effects of interventions on the ability to estimate the body circumference.

## 5 Conclusions

This study identified neural responses related to reduced body-image disturbance. Participants who demonstrated such a reduction also exhibited changes in brain activation. Decreased perceptual disturbance was associated with increased activation of bodily visuospatial processing regions, including the left superior parietal lobule, bilateral superior occipital gyri, and right cuneus. On the other hand, reduced affective disturbance was associated with changes in neural responses in socio-cognitive processing regions, including decreased activation in the right temporoparietal junction and an increase in functional connectivity from the left extrastriate body area to the right precuneus. These results partially support the results of our previous studies investigating the neural responses associated with each type of body-image disturbance, implying significant differences in neural mechanisms between reduction and expression of body-image disturbance. Although we observed neural responses related to the effects of interventions at the individual level, we did not detect significant collective behavioral improvements. In conclusion, our results enhance our understanding of the neural mechanisms underlying body-image disturbance and could inform the development of treatment and prevention methods for eating disorders.

## Data availability statement

The raw data supporting the conclusions of this article will be made available by the authors, without undue reservation.

## Ethics statement

The studies involving humans were approved by The Ethics Committee of Tohoku University Graduate School of Medicine. The studies were conducted in accordance with the local legislation and institutional requirements. The participants provided their written informed consent to participate in this study.

## Author contributions

YH: Conceptualization, Data curation, Formal analysis, Funding acquisition, Investigation, Methodology, Project administration, Software, Visualization, Writing – original draft, Writing – review & editing. KO: Investigation, Writing – review & editing. RI: Investigation, Writing – review & editing. YD: Investigation, Writing – review & editing. RN: Investigation, Writing – review & editing. MS: Conceptualization, Funding acquisition, Investigation, Methodology, Project administration, Resources, Supervision, Writing – original draft, Writing – review & editing.
