# Supplementary material for: Reduced body-image disturbance by body-image interventions is associated with neural-response changes in visual and social processing regions: a preliminary study
Source: Front Psychiatry. 2024 Mar 6;15:1337776. doi: 10.3389/fpsyt.2024.1337776 (PMC10951070; doi:10.3389/fpsyt.2024.1337776)
Supplement: Supplementary file 1 [file Table_1.docx]

Supplementary Material

Reduced body-image disturbance by body-image interventions is associated with neural-response changes in visual and social processing regions: A preliminary study

Yumi Hamamoto^1,2,3*^, Kentaro Oba^1^, Ryo Ishibashi^1^, Yi Ding^1,3,4^, Rui Nouchi^1^, Motoaki Sugiura^1,5^

^1^Institute of Development, Aging and Cancer, Tohoku University, Sendai, Japan

^2^Department of psychology, Northumbria University, Newcastle upon Tyne, UK

^3^Japan Society for the Promotion of Science, Tokyo, Japan

^4^School of Medicine, Tohoku University, Sendai, Japan

^5^International Research Institute of Disaster Science, Tohoku University, Sendai, Japan

*** Correspondence:**Yumi Hamamoto
[yumi.hamamoto.q2@gmail.com](mailto:yumi.hamamoto.q2@gmail.com)

**Supplementary table 1. Transformed scores of Eating Disorder Inventory 2 before and after interventions**

|  | Mean (SD) | | | | | |
| --- | --- | --- | --- | --- | --- | --- |
|  | All participants | | Mirror exposure | | Mental imagery | |
|  | Pre | Post | Pre | Post | Pre | Post |
| Total score | 17.0 (11.0) | 15.32 (10.4) | 12.4 (8.46) | 11.7 (7.69) | 21.7 (11.5) | 18.9 (11.6) |
| Body dissatisfaction | 12.0 (7.34) | 11.5 (6.98) | 9.14 (6.13) | 9.43 (6.19) | 14.8 (7.56) | 13.5 (7.36) |
| Drive for thinness | 5.07 (4.70) | 3.86 (4.66) | 3.21 (3.31) | 2.29 (2.76) | 6.93 (5.24) | 5.43 (5.67) |

Total score means the sum of body dissatisfaction and drive for thinness scores. The analysis of covariance showed that there was no significant main effect of interventions (total score: *η_p_^2^* [partial *η^2^*] = 0.008, *p* = 0.09, body dissatisfaction: *η_p_^2^* = 0.001, *p* = 0.59, drive for thinness: *η_p_^2^* = 0.02, *p* = 0.12). There was no significant interaction effect (total score: *η_p_^2^* [partial *η^2^*] = 0.003, *p* = 0.28, body dissatisfaction: *η_p_^2^* = 0.004, *p* = 0.40, drive for thinness: *η_p_^2^* = 0.001, *p* = 0.71).

Note: SD = standard deviation.
